# Multivariate Analysis of Factors Affecting Presence and/or Agenesis of Third Molar Tooth

**DOI:** 10.1371/journal.pone.0101157

**Published:** 2014-06-26

**Authors:** Mohammad Khursheed Alam, Muhammad Asyraf Hamza, Muhammad Aizuddin Khafiz, Shaifulizan Abdul Rahman, Ramizu Shaari, Akram Hassan

**Affiliations:** 1 Orthodontic Unit, School of Dental Science, Universiti Sains Malaysia, Kota Bharu, Kelantan, Malaysia; 2 Oral and Maxillofacial Surgery Unit, School of Dental Science, Universiti Sains Malaysia, Kota Bharu, Kelantan, Malaysia; 3 Periodontology Unit, School of Dental Science, Universiti Sains Malaysia, Kota Bharu, Kelantan, Malaysia; University of North Carolina at Chapel Hill, United States of America

## Abstract

To investigate the presence and/or agenesis of third molar (M3) tooth germs in orthodontics patients in Malaysian Malay and Chinese population and evaluate the relationship between presence and/or agenesis of M3 with different skeletal malocclusion patterns and sagittal maxillomandibular jaw dimensions. Pretreatment records of 300 orthodontic patients (140 males and 160 females, 219 Malaysian Malay and 81 Chinese, average age was 16.27±4.59) were used. Third-molar agenesis was calculated with respect to race, genders, number of missing teeth, jaws, skeletal malocclusion patterns and sagittal maxillomandibular jaw dimensions. The Pearson chi-square test and ANOVA was performed to determine potential differences. Associations between various factors and M3 presence/agenesis groups were assessed using logistic regression analysis. The percentages of subjects with 1 or more M3 agenesis were 30%, 33% and 31% in the Malaysian Malay, Chinese and total population, respectively. Overall prevalence of M3 agenesis in male and female was equal (*P*>0.05). The frequency of the agenesis of M3s is greater in maxilla as well in the right side (*P*>0.05). The prevalence of M3 agenesis in those with a Class III and Class II malocclusion was relatively higher in Malaysian Malay and Malaysian Chinese population respectively. Using stepwise regression analyses, significant associations were found between Mx (*P*<0.05) and ANB (*P*<0.05) and M3 agenesis. This multivariate analysis suggested that Mx and ANB were significantly correlated with the M3 presence/agenesis.

## Introduction

Tooth agenesis is the congenital lack of one or more of the deciduous or permanent teeth – the one not erupted in the oral cavity, and also not visible in a radiograph, is one of the most frequent human dental anomales [1]. The third molar (M3) is a tooth characterized by the variability in the time of its formation, its widely varying crown and root morphology, and its varying presence or absence in the oral cavity [2]. Agenesis of one or more permanent teeth is a common anomaly in man and many reports on M3 agenesis have been published for different populations over the last 50 years [3]–[24].

The wide range of prevalence of this anomaly might be attributed to the differences in the methods of sampling and examination, age and sex distribution, and racial origin of the subjects. 7.

M3 agenesis has been associated with dental numeric and structured variations. Garn et al. [25] have suggested that when a M3 is absent, agenesis of the remaining teeth is 13 times more likely. M3 is undoubtedly the most common dental reduction with up to 50% of some groups affected [16].

Investigators and clinicians, especially orthodontists, believe that an increase in agenesis of permanent teeth is related to dentofacial development, and development of malocclusion. There have been many debates for years on whether there is a relationship between third molars and crowding [25], [26], Clinicians, especially orthodontist should consider the entire dentition including the presence or absence of the third molars because it relates with posterior crowding. A limited number of studies have been carried out to evaluate the relationship between M3 agenesis and different skeletal malocclusion patterns [18], [20], [27], sagittal jaw dimensions [27], [28] and craniofacial morphology [27].

We have, therefore, given particular attention to the subjects of presence and/or agenesis of M3. As per our concern no studies have been carried out to evaluate the relationship between presence and/or agenesis of M3 with different skeletal malocclusion patterns and sagittal maxillomandibular jaw dimensions in Malaysian Malay and Chinese polpulation. On the basis of these facts, the aim of this study was to -.

1. investigate the presence and/or agenesis of third molar tooth germs in orthodontics patients in Malaysian Malay and chinese population.
2. examine the relationship between presence and/or agenesis of M3 and different sagittal skeletal malocclusions.
3. examine the relationship between presence and/or agenesis of M3 and different vertical patterns of the skeletal malocclusions.
4. to determine the existence of any relation between the presence and/or agenesis of third molar tooth germs and sagittal maxillomandibular jaw dimensions.

## Materials and Methods

All participants provide their written informed consent (One of the parents, either father and/or mother gave written consent for the adolescent subjects). This study was approved by the Ethical Committee of the Hospital Universiti Sains Malaysia (HUSM) [FWA Reg. No: 00007718; IRB Reg. No: 00004494], which complies with the Declaration of Helsinki. This study was designed and conducted according to the guidelines of Strengthening the Reporting of Observational studies in Epidemiology (STROBE), and we applied the STROBE checklist in the preparation of this manuscript [28].

Power and sample size software calculated the sample size with a power of 80%; the alpha was 0.05.

Sample size calculation:
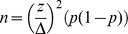



n = number of sample.

z = z-score at 95% confidence interval = 1.96.

Δ = precision.

p = proportion taken from previous published study [18].

Considering the available resources, number of sample, n = 270.

Also, considering the missing data 10% of sample is added, so the sample size is 297.

Finally three hundred patients (140 males and 160 females, average age was 16.27±4.59) were selected for this study from the orthodontic unit of Hospital Universiti Sains Malaysia.

### Inclusion criterion

1. the patients had not received any orthodontic treatment,
2. the patients had not undergone surgical removal or extraction of one or more M3s.

### Exclusion criterion

1. subjects with congenital deformities,
2. radiographs of poor quality.

The digital images were investigated (Orthopantomogram [OPG]) measured (Lateral cephalograms) using Romexis software (Planmeca, Finland) operating with computer (DX2810 Microtower PC, HP Compaq, US) and 17-inch monitor screen (LE1711 LCD monitor, HP Compaq, US).

### OPG investigation

Panoramic radiographs taken at the initial examination were used to determine the presence of M3 germs. In cases in which it was impossible to judge the presence of M3 germs from panoramic radiographs taken at the initial examination, were also excluded.

### Cephalometric analysis

**Figure 1:**
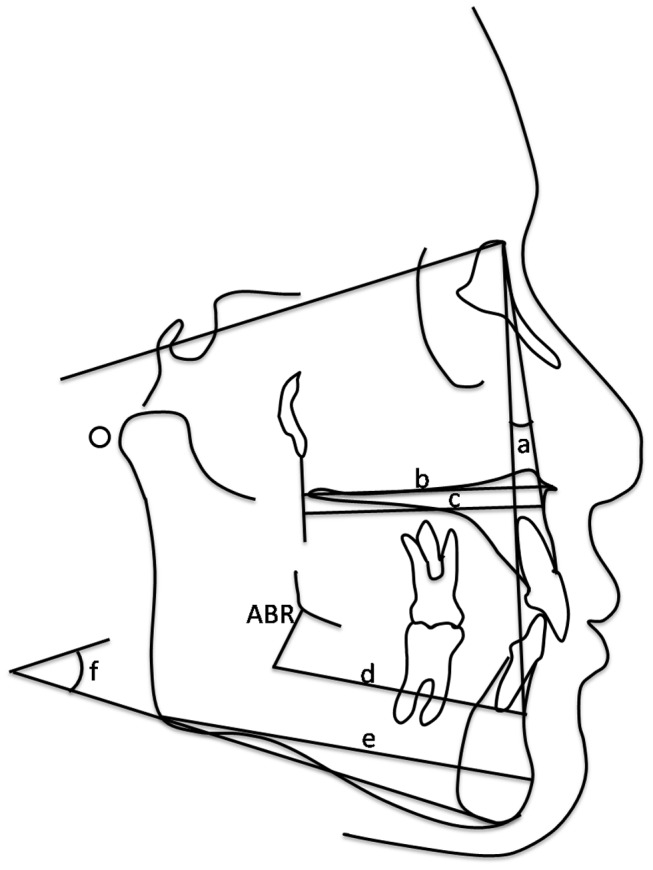
Angular cephalometric measurements relating to sagittal and vertical malocclusion and linear cephalometric measurements relating to sagittal jaw dimensions (descriptions are in Table 1).

Lateral cephalograms also taken at the initial examination were used to measure linear and angular cephalometric variables (**Table 1** **and** **Figure 1**). For the sagittal skeletal malocclusions, skeletal Class I (1° and 5°), Class II (>5°) and Class III (<1°) using the measurements of the ANB angle was used. SN-GoMe angle was used for classification of vertical patterns of the skeletal malocclusions as being normal (27°–37°), hypo-divergent (<27°), and hyper-divergent (>37°).

**Table 1:** Cephalometrics angular and linear measurements.

Variable	Measuring unit	Description
(a) ANB	degree	Angle formed by a line joining NA and NB
(b) ANS-PNS	mm	Anteroposterior length of the nasal floor
(c) A-Ptm (Mx)	mm	Anteroposterior length of the maxillary basal bone
(d) ABR-B (Mn)	mm	anteroposterior length of the mandibular basal bone (ABR: cross point between occlusal plane and anterior edge of the ramus)
(e) Go-Pog	mm	anteroposterior length of the corpus
(f) SN-MP	degree	Angle formed by SN plane and mandibular plane.

### Statistical Analysis

The data were verified and analysed statistically using IBM SPSS Statistics Version 20.0 with confidence level set at 5% (P<0.05) to test for significance.

Randomly selected 50 OPG were evaluated by another researcher 4 weeks after the initial survey to determine the reliability of diagnosis of the M3 agenesis. The kappa statistics has been used to determine intra- and interexaminer agreements. There was 100% intra- and interexaminer agreement between the investigators. To determine the errors associated with digitizing and measurements, 50 radiographs were selected randomly. All procedures such as landmark identification, tracing, and measurements were repeated 4 weeks after the first examination by the same investigator. Intraclass correlation coefficients were performed to assess the reliability of the measurements and the coefficients of reliability of the measurements were between 0.93 and 0.99.

Dahlberg’s formula was used to determine the method-error of cephalometric measurements, which did not exceed 0.38 mm for the linear variables, 0.63 degree for the angular variables. The combined error for any of the variable was small and considered to be within acceptable limit [29]. Dalhberg’s formula: **ME = √Σ(x1−x2)^2^/2n**. Where x1 is the first measurement, x2 the second measurement and n the number of repeated records [29]. The M3 presence and/or agenesis were calculated with respect to race, genders, number of missing teeth, jaws, and skeletal malocclusion patterns. The Pearson chi-square test was performed to determine potential differences in the distribution of M3 agenesis when stratified according to the above parameters. The existence of significant differences between the presence and/or agenesis of third molar tooth germs and sagittal maxillomandibular jaw dimensions was analyzed by ANOVA for one factor and the Scheffe test for multiple comparisons.

Logistic regression analysis was performed using the dichotomous dependent variable, M3 presence vs agenesis groups. Both crude and backward stepwise logistic regression analyses were done to determine which factors associated with the M3 presence/agenesis [30].

## Results

Presence and/or agenesis of third molar tooth germs in orthodontics patients in Malaysian Malay and Chinese population:

### Inter races disparities

**Figure 2:**
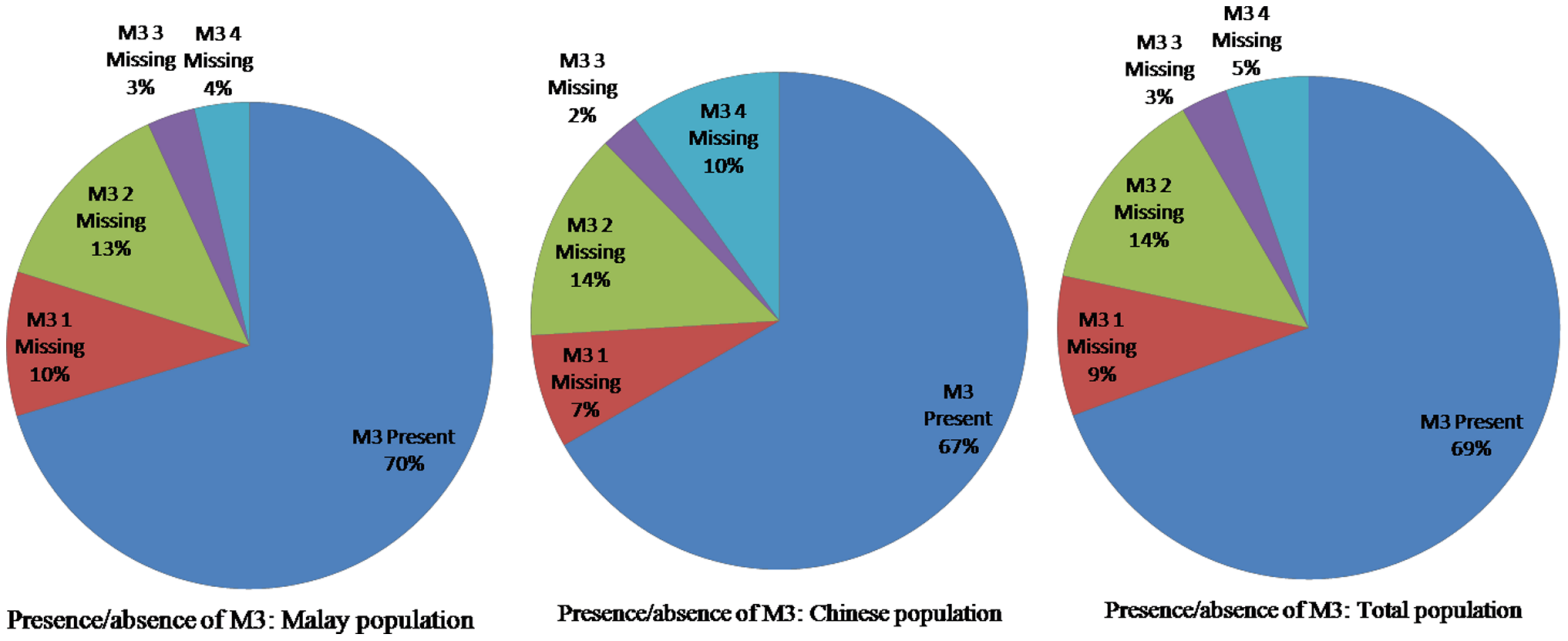
Distribution of Subjects with M3 Presence/Agenesis in Malysian Malay and Chinese.

**Figure 2** shows the percentages of subjects with all 4 M3 present and with 1 or more of the M3 missing. The percentages of subjects with all M3 were 70%, 67% and 69% in the Malaysian Malay, Chinese and total population, respectively. Therefore, the overall prevalence of M3 agenesis was 31% in this orthodontic population. The difference between the groups was not significant. Among the patients with M3 agenesis in Malaysian Malay population, the prevalence of patients with one, two, three, or four missing tooth/teeth were 10%, 13%, 3%, and 4%, respectively. In Malaysian Chinese population, the prevalence of patients with one, two, three, or four M3 missing tooth/teeth were 7%, 14%, 2%, and 10%, respectively. There is no significant difference in the occurrence of M3 agenesis between the numbers of M3s.

### Inter sexes disparities

**Figure 3:**
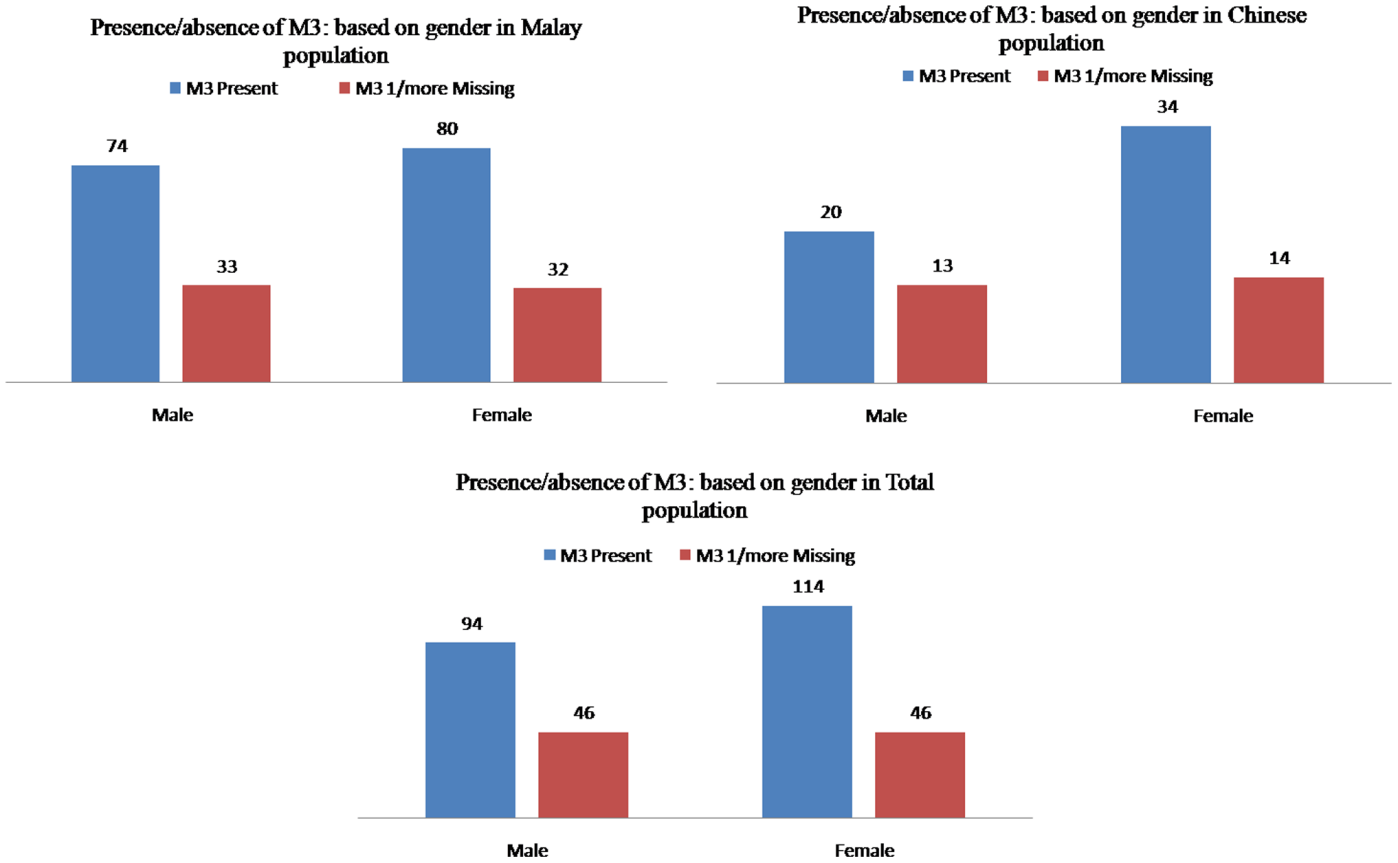
Distribution of Subjects with M3 Presence/Agenesis in male and female.

**Figure 3** shows overall prevalence of M3 agenesis in male and female was equal. There was no significant gender difference.

### Inter side (right and left) and jaw (maxilla, mandible and both)

**Figure 4:**
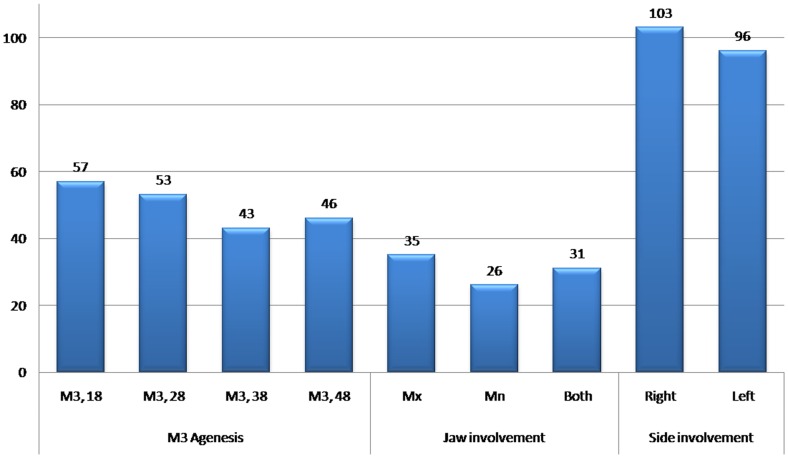
Distribution of Subjects in Total (Malaysian Malay and Chinese) population With M3 Agenesis, Jaw and side involvement.

The difference between the groups in relation to side and jaw involvement was not significant (data not shown). The distribution of M3s present in the maxilla and the mandible or both and on the right and the left side is depicted in **Figure 4**. Although the frequency of the agenesis of M3s is greater in maxilla as well in right side, the χ2 statistic revealed no significant relationship, indicating that there is no correlation between the frequencies of M3 existence.

### Inter sagittal skeletal malocclusions disparities

**Figure 5:**
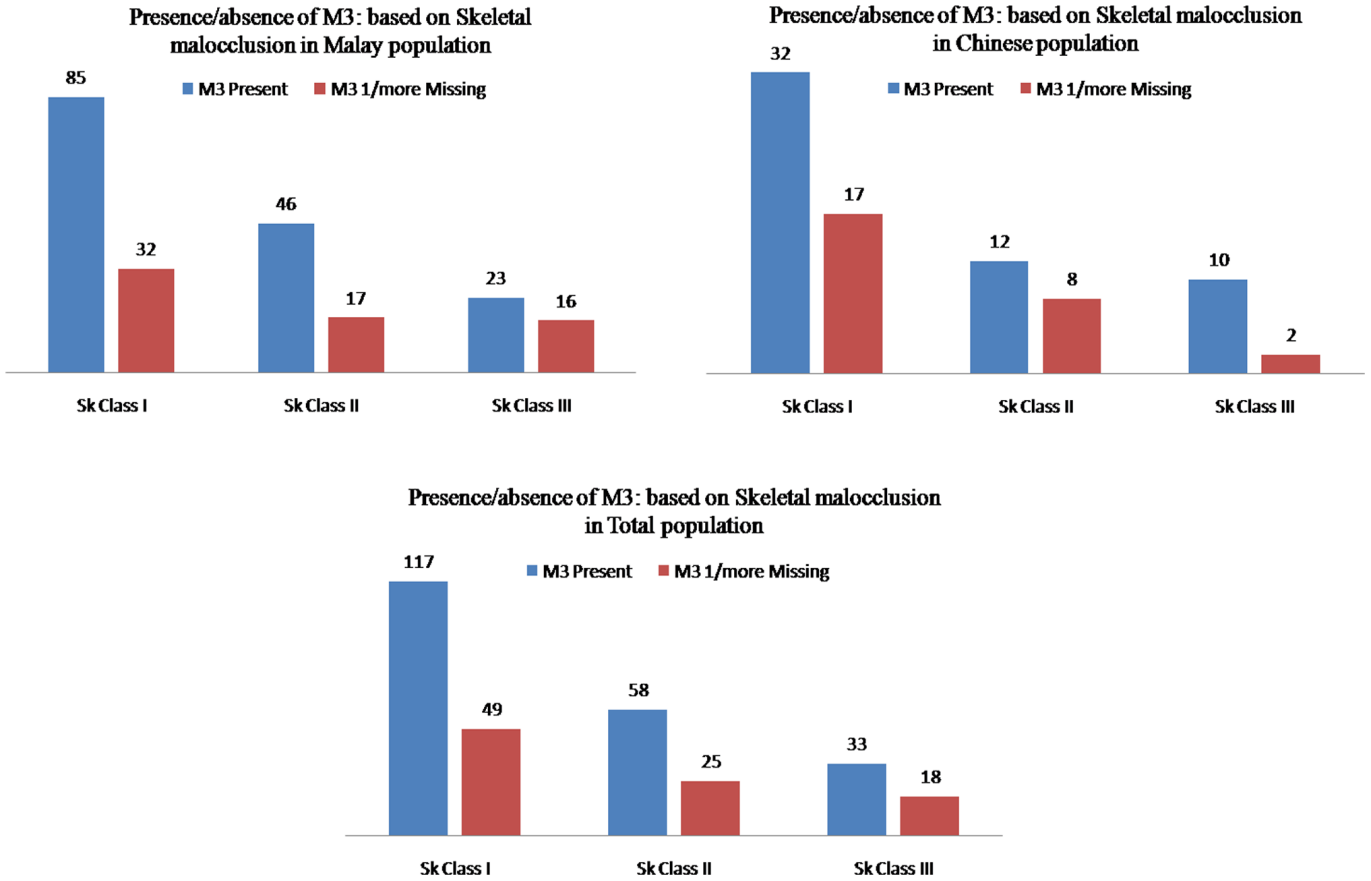
Distribution of Subjects with M3 Presence/Agenesis in Sagittal Skeletal Malocclusions.

**Figure 5** shows the prevalence of M3 agenesis (total population) in those with a Class I, Class II, or Class III malocclusion was 29.5%, 30.1%, and 35.3%, respectively. The prevalence of M3 agenesis in those with a Class III malocclusion (41% in Malaysian Malay and 35% in total population) was relatively higher than in those with a Class I or a Class II. However in Malaysian Chinese population, the prevalence of M3 agenesis in those with a Class II malocclusion (40%) was relatively higher than in those with a Class I or a Class III. The difference between the groups was not significant.

### Inter vertical patterns of the skeletal malocclusions disparities

**Figure 6:**
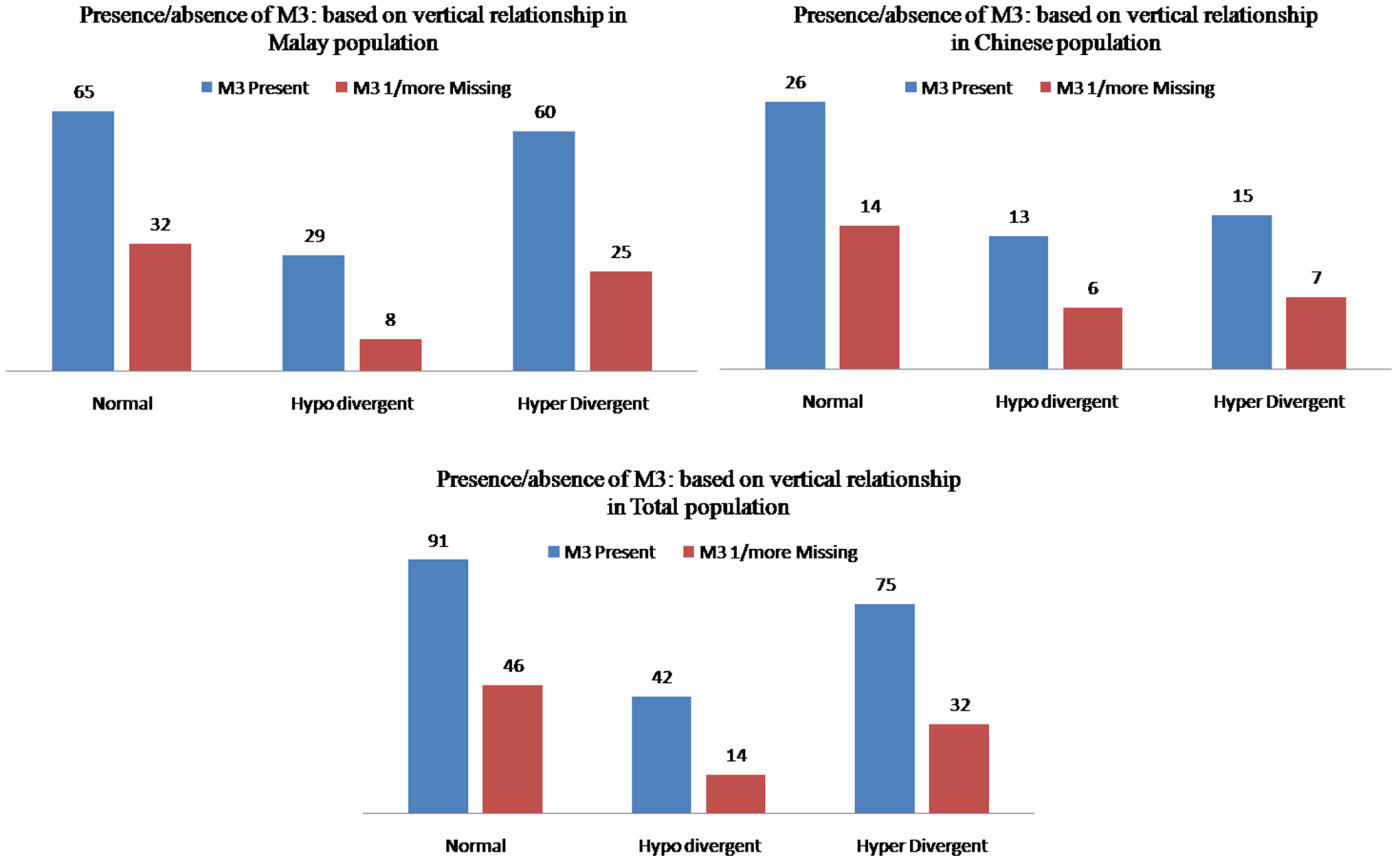
Distribution of Subjects with M3 Presence/Agenesis in Vertical Skeletal Malocclusions.

**Figure 6** shows the prevalence of M3 agenesis (total population) in those with a normal, hypo-divergent and hyper-divergent groups was 33.6%, 25%, and 29.9%, respectively. The difference between the groups was not significant.

### Inter sagittal maxillomandibular jaw relationships disparities

Table 2, 3 and 4 shows the results obtained by linear and angular cephalometric measurement between 4 M3 present and with 1 or more of the M3 missing in Malaysian Malay, Chinese and total population. The frequency of M3 agenesis (4 M3s agenesis) increased with an increasing Mx (Table 3 and 4) and with a decreasing Mn (Table 2, 3 and 4). Significant differences between the groups were not detected.

**Table 2:** Linear and Angular Cephalometric Measurements in a Malay population With M3 Presence/Agenesis.

Variables	M3 Present		M3, 1 missing		M3, 2 missing		M3, 3 missing		M3, 4 missing	
	Mean	SD	Mean	SD	Mean	SD	Mean	SD	Mean	SD
ANS-PNS	51.18	5.06	50.38	3.74	52.59	5.30	50.04	4.82	50.83	4.13
Go-Pog	70.28	6.31	70.17	7.40	69.72	4.89	67.11	7.34	68.96	5.32
Mx	46.82	4.65	48.17	7.62	47.21	4.26	45.84	4.80	46.55	3.06
Mn	48.91	9.56	49.35	6.39	47.34	7.97	45.99	5.52	45.69	4.21
ANB	4.09	2.68	3.05	2.84	3.69	3.93	2.86	2.54	3.75	1.67
SN-MP	35.86	6.74	35.33	6.07	37.66	6.16	36.29	8.44	35.63	4.21

ANOVA applying Post Hoc Scheffe test * P<0.05. The groups did not show significant differences with any other.

**Table 3:** Linear and Angular Cephalometric Measurements in a Chinese population With M3 Presence/Agenesis.

Variables	M3 Present		M3, 1 missing		M3, 2 missing		M3, 3 missing		M3, 4 missing	
	Mean	SD	Mean	SD	Mean	SD	Mean	SD	Mean	SD
ANS-PNS	53.95	4.94	58.68	5.74	53.21	4.12	57.85	0.49	54.78	8.19
Go-Pog	73.83	7.02	77.27	7.92	75.05	4.67	78.25	3.04	73.99	10.97
Mx	48.48	4.12	52.88	5.20	48.07	3.17	50.85	1.77	50.24	7.11
Mn	48.30	7.61	50.20	7.11	46.03	7.42	50.05	1.34	45.28	7.24
ANB	3.83	2.41	5.67	1.37	3.55	1.69	3.50	2.12	3.88	2.36
SN-MP	34.59	6.85	32.33	4.63	34.64	5.97	34.50	2.12	34.25	5.70

ANOVA applying Post Hoc Scheffe test * P<0.05. The groups did not show significant differences with any other.

**Table 4:** Linear and Angular Cephalometric Measurements in Total (Malaysian Malay and Chinese) population With M3 Presence/Agenesis.

Variables	M3 Present		M3, 1 missing		M3, 2 missing		M3, 3 missing		M3, 4 missing	
	Mean	SD	Mean	SD	Mean	SD	Mean	SD	Mean	SD
ANS-PNS	51.90	5.17	52.22	5.43	52.76	4.96	51.78	5.41	52.80	6.59
Go-Pog	71.20	6.67	71.74	7.95	71.19	5.34	69.59	8.10	71.48	8.72
Mx	47.25	4.57	49.22	7.34	47.45	3.97	46.96	4.74	48.39	5.62
Mn	48.75	9.08	49.54	6.42	46.98	7.75	46.89	5.13	45.48	5.73
ANB	4.02	2.61	3.63	2.79	3.65	3.44	3.00	2.35	3.81	1.97
SN-MP	35.53	6.78	34.67	5.84	36.83	6.18	35.89	7.39	34.94	4.89

ANOVA applying Post Hoc Scheffe test * P<0.05. The groups did not show significant differences with any other.

### Crude logistic regression analysis

**Table 5** shows the results of the crude logistic regression analysis that estimated the associations between various factors (independent variable) and M3 presence/agenesis (dependent variable). Odds ratio, 95% confidence interval, and p value for the various factors are presented. No significant associations were found among various factors (age, sex, race, ANSPNS, GO-POG, Mx, Mn, ANB, SN-MP) with the M3 presence/agenesis.

**Table 5:** Crude logistic regression analysis: M3 presence vs. agenesis group.

	S.E.	Exp(B)	95% Confidence Interval		*P - value*
			Lower	Upper	
Age	0.029	1.030	0.973	1.089	0.310
Gender	0.251	0.825	0.504	1.348	0.442
Race	0.278	1.185	0.687	2.044	0.543
ANS-PNS	0.024	1.023	0.976	1.071	0.348
GO-POG	0.019	1.001	0.965	1.038	0.960
Mx	0.026	1.035	0.985	1.088	0.174
Mn	0.015	0.982	0.953	1.012	0.226
ANB	0.046	0.945	0.863	1.035	0.221
SN-MP	0.019	1.006	0.969	1.044	0.766

S.E. = Standard error, Exp(B = Odds ratio), * P<0.05.

### Stepwise logistic regression analysis

**Table 6** shows the results of the stepwise logistic regression analysis that estimated the associations between various factors and M3 presence/agenesis. Significant associations were found among various factors, Mx (P<0.05) and ANB (P<0.05) were significantly correlated with M3 presence/agenesis.

**Table 6:** Adjusted logistic regression analysis (stepwise regression analysis: backward method): M3 presence vs. agenesis group.

	S.E.	Exp(B)	95% Confidence Interval		*P - value*
			Lower	Upper	
Mx	0.033	1.084	1.015	1.158	0.016*
Mn	0.021	0.964	0.924	1.005	0.082
ANB	0.054	0.887	0.798	0.985	0.025*

S.E. = Standard error, Exp(B) = Odds ratio), * P<0.05.

Variables entered on step 1: age, sex, race, ANSPNS, GO-POG, Mx, Mn, ANB and SN-MP.

## Discussion

**Figure 7:**
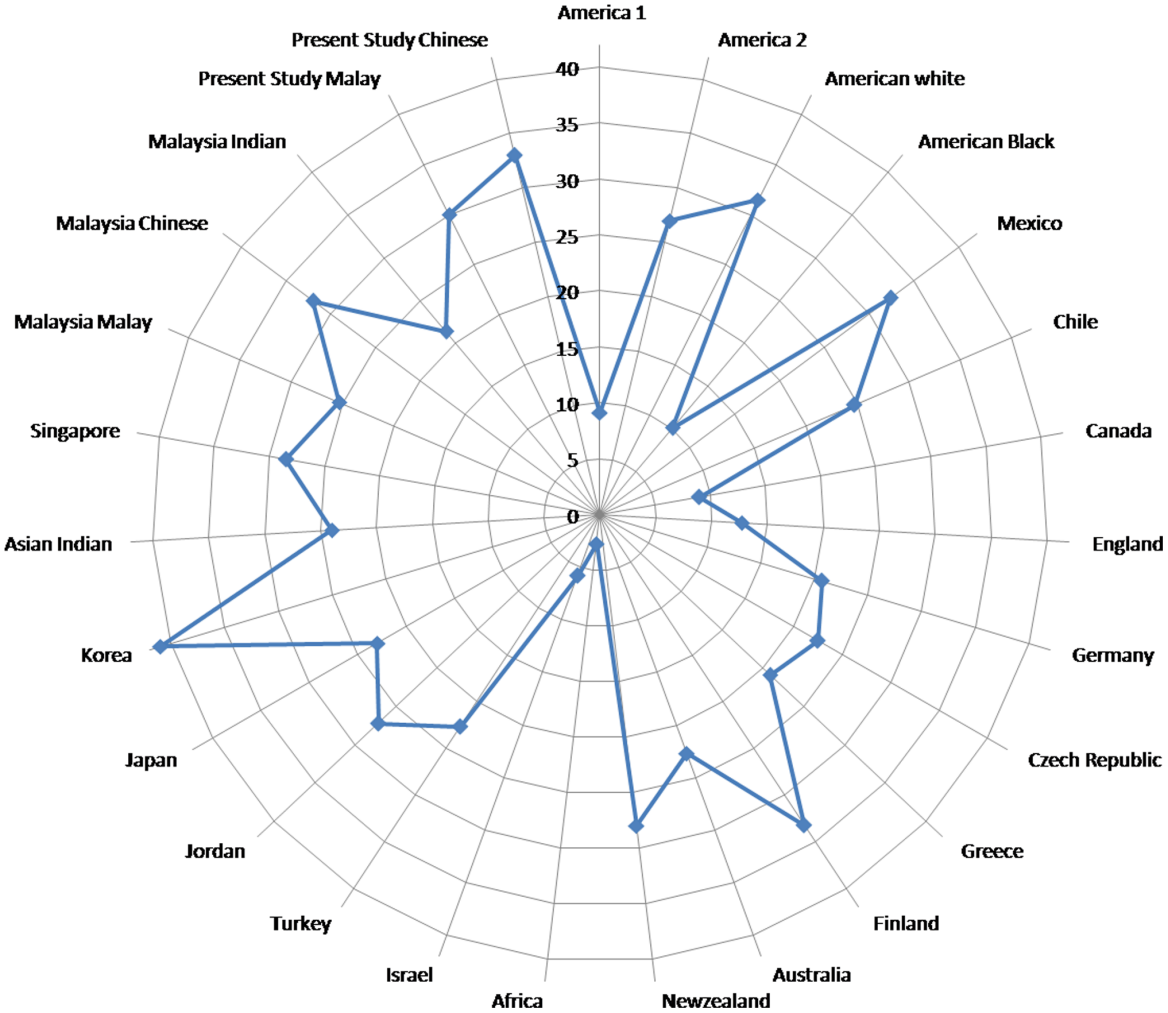
Global Distributions of prevalence of M3 Agenesis.

In the present study based on panoramic radiographs, we attempted to determine the prevalence of M3 presence and/or agenesis in a sample of orthodontic patients from Malaysian Malay and Chinese. We found that 31% of the subjects had 1 or more M3s agenesis, These results indicate that about one third of the patients had 1 or more M3s agenesis, which is close to the frequency of 31.5% reported by Harris and Clark [16] for American white subjects, 32.4% reported by Rosario and Gonzalez [6] for Mexican subjects, 33.2% reported by Eloma and Eloma [13] for Finland subjects and 32% reported by Jacob et al. [24] for Malaysian Chinese subjects. Global distributions of the prevalence of M3 agenesis were shown in **Figure 7** [3]–[24]. These racial differences are interesting and suggest that some polygenetic inheritance on formation of M3 germs may differ among populations and races as well as may be due to differences in sample sizes.

In our study, the order of frequency for the M3 agenesis is two, one, four, and three M3s in Malaysian Malay subjects [24]. In Malaysian Chinese subjects, the order of frequency for the M3 agenesis is two, four, one, and three M3s. In total population, the order of frequency for the M3 agenesis is two, one, four, and three M3s [24]. The order of frequency for M3 agenesis in this study is incompatible with the reports published by those authors [16], [18], [22], [23].

Intersexual comparisons revealed no significant differences in the incidence of M3 agenesis, despite the higher percentage noted for males than that for females (32.9% and 28.8%, respectively). The male predominance of M3 agenesis agrees with the results reported by Hattab et al. [19] but differs from data reported by Jacob et al. [24], Barka et al. [12] and Sandhu and Kaur [22]. On the other hand, all the above-mentioned authors concluded that intersexual differences were not statistically significant, which is in agreement with our results. No significance was also reported for the Turkish [18], Chinese [23] and Japanese [20] populations.

The order of frequency for the M3 agenesis is 18, 28, 48 and 38 is found in the present study. However Jacob et al. [24] found 18, 28, 38 and 48. Studies performed in different populations demonstrate that the majority of missing M3s were located in the maxilla, with the differences being statistically significant [20], [23]. Hattab et al. [19], Sandhu and Kaur [22], Barka et al. [12] and Jacob et al. [24] also reported that congenitally missing M3s showed a greater predilection for the maxilla over the mandible. The reason there may be a disparity in M3 agenesis in the maxilla and mandible is also not clear [31]. Besides, in the present study, no significant difference was found between the frequencies of at least one M3 missing in the maxilla and the mandible.

In this study, crude logistic regression analysis was used to estimate associations between each factors (age, sex, race, ANSPNS, GO-POG, Mx, Mn, ANB, SN-MP) and M3 presence/agenesis. Stepwise logistic regression analysis was used to explore the associations between precise factors (among various factors) and M3 presence/agenesis. Stepwise logistic regression analysis is used in the exploratory phase of research [30]. Backward stepwise regression appears to be the preferred method of exploratory analyses, in which the analysis begins with a full model and variables are eliminated one by one using the largest p value [30]. The final model is the last step model, in which eliminating another variable would not improve the model significantly [30].

Sanchez et al. [27] hypothesized that agenesis of wisdom teeth is not related with any particular craniofacial morphology which is in agreement with the results of the present study except for the Mx. Present study revealed presence and/or agenesis of M3 depends significantly on sagittal skeletal malocclusions (ANB), and sagittal jaw dimensions (Mx) in the exploratory phase of analysis. Perhaps the difference in results could be linked to racial differences. Such differences are interesting; the reasons stated that some polygenetic inheritance on formation of M3 germs may be related to genes that control maxillary and/or mandibular dimensions. Present study also revealed presence and/or agenesis of M3 do not depends significantly on vertical patterns of the skeletal malocclusions. However, Sanchez et al. [27] concluded as maxillary M3 agenesis are related to reduced mandibular plane angles and mandibular M3 agenesis showed a diminished lower third and mandibular morphology characteristic of brachyfacial patterns.

Results of the present study are the evidence for the prevalence of M3 agenesis in those with a Class III malocclusion in Malaysian Malay population was relatively higher. However in Malaysian Chinese population, the prevalence of M3 agenesis in those with a Class II malocclusion was relatively higher. On the other hand Celikoglu and Kamak [18] and Kajii et al. [30] found that the prevalence of Class III subjects who had all four M3s was lower than that of subjects with Class II malocclusions. Celikoglu and Kamak [18] stated that the vertical patterns of the skeletal malocclusions for M3 agenesis patients showed with an order of prevalence in hyper-divergent, normal, and hypo-divergent groups. On the other hand, Sanchez et al. [27] found that agenesis of maxillary M3s was related to a reduced mandibular plane angle. In our study, we found that the order of prevalence is normal, hyper-divergent and hypo-divergent groups without any significant difference [18].

In a study of a group of Spanish, Sanchez et al. [27] evaluated the relationship of third molar agenesis to craniofacial morphology. They reported that subjects with bilateral maxillary agenesis, bilateral mandibular agenesis and control group (All M3 present) have no significant association with sagittal jaw dimensions which is in agreement with our finding. However, Kajii et al. [31] reported that subjects with bilateral maxillary agenesis of the M3 were significantly associated with maxilla and no significant association was shown between the sagittal dimension of the mandible and M3 agenesis.

In a prospect study, polygenetic inheritance on formation of M3 germs and the genes that control maxillary and/or mandibular dimensions and of craniofacial maturation need to be studied.

## Conclusion

- The present results showed that in this orthodontic population from Malaysian Malay and Chinese, M3 agenesis accounted for 30% and 33% respectively.
- The frequency of M3 agenesis was found greater in the maxilla.
- These results revealed that Presence and/or agenesis of M3 do not depends significantly on age, sex, race, side, jaw involvement and vertical skeletal malocclusions.
- This multivariate analysis suggested that increasing Mx and ANB were significantly correlated with the M3 agenesis in this orthodontic population.
